# A new species of the genus *Gonatopus* Ljungh from the USA (Hymenoptera, Dryinidae)

**DOI:** 10.3897/zookeys.747.24399

**Published:** 2018-03-29

**Authors:** Adalgisa Guglielmino, Massimo Olmi, Alessandro Marletta, Stefano Speranza

**Affiliations:** 1 Department of Agriculture and Forestry Sciences (DAFNE), University of Tuscia, Viterbo, Italy; 2 Tropical Entomology Research Center, Viterbo, Italy; 3 Department of Biological, Geological and Environmental Sciences, Animal Biology section, University of Catania, Catania, Italy

**Keywords:** Chrysidoidea, Florida, Gonatopodinae, key, Nearctic region, taxonomy

## Abstract

A new species of *Gonatopus* Ljungh, 1810 is described from the USA, Florida: *G. jacki* **sp. n**. Morphologically, the new species is similar to *G. ashmeadi* Kieffer, 1905 and *G. agropyrus* Fenton, 1921, but it is distinguished by the different shape of the mesoscutum (very slender in *G. jacki*; broader in *G. ashmeadi* and *G. agropyrus*). Published identification keys to the Nearctic species of *Gonatopus* are modified to include the new species.

## Introduction

Based on all known host records, Dryinidae (Hymenoptera, Chrysidoidea) are parasitoids of Auchenorrhyncha (Hemiptera) (Guglielmino et al. 2013). However, the biology of this group of small wasps is still poorly known (Guglielmino et al. 2006, 2008, 2015). *Gonatopus* Ljungh, 1810 is a genus that is present in all zoogeographical regions (Olmi 1984; Guglielmino and Virla 1998; Guglielmino and Bückle 2003, 2010; Xu et al. 2013; Olmi and Virla 2014; Olmi and Xu 2015). In total 441 species have been described from all continents (Guglielmino and Olmi 2014; Olmi and Xu 2015) and the genus was revised at world level by Olmi (1984, 1991), and more recently in the Oriental, Neotropical and Eastern Palaearctic regions by Xu et al. (2013), Olmi and Virla (2014) and Olmi and Xu (2015),

The species of *Gonatopus* inhabiting the Nearctic region were studied by Olmi (1984, 1987, 1992, 1993a, 1993b, 1995, 2003). More recently, Olmi and Guglielmino (2013) described one further new species from the USA, Arkansas (*Gonatopus rileyi* Olmi & Guglielmino). In total, 51 *Gonatopus* species have been described from the Nearctic region (Olmi 1984, 1987, 1992, 1993a, 1993b, 1995, 2003; Olmi and Guglielmino 2013).

*Gonatopus* species are parasitoids of leafhoppers and planthoppers belonging to the Acanaloniidae, Cicadellidae, Delphacidae, Dictyopharidae, Flatidae, Issidae, Lophopidae, Meenoplidae, Tropiduchidae (Guglielmino et al. 2013). As in almost all dryinids, females of *Gonatopus* have a chelate protarsus. Chelae are used to capture and restrain the host during ovipositions and hostfeeding (Olmi 1984, 1994).

In 2015, additional specimens of *Gonatopus* from the USA were examined and the new species found is described herein.

## Materials and methods

The descriptions follow the terminology used by Olmi (1984), Olmi and Guglielmino (2010), and Olmi and Virla (2014). The measurements reported are relative, except for the total length (head, except antennae, to abdominal tip), which is expressed in millimeters. In the descriptions, **POL** is the distance between the inner edges of the two lateral ocelli; **OL** is the distance between the inner edges of a lateral ocellus and the median ocellus; **OOL** is the distance from the outer edge of a lateral ocellus to the compound eye; **OPL** is the distance from the posterior edge of a lateral ocellus to the occipital carina; and **TL** is the distance from the posterior edge of an eye to the occipital carina. The term “metapectal-propodeal complex” is here used in the sense of Kawada et al. (2015). It corresponds to the term “propodeum” *sensu* Olmi (1984, 1994), Xu et al. (2013), Olmi and Virla (2014), and Olmi and Xu (2015).

The types of all Nearctic and Neotropical species of *Gonatopus* have been previously examined by the authors.

The material studied in this paper is deposited in the Department of Agriculture and Forestry Sciences, University of Tuscia, Viterbo, Italy (**MOLC**).

The description of the new species is based on the study of a single specimen. The authors are aware that descriptions of new taxa should normally be based on more individuals. However, Dryinidae are so rare that it is uncommon to collect more than one specimen of each species. In addition, on the basis of the experience and knowledge of the authors, the new species is sufficiently delimited by unique characters to justify its description.

## Taxonomy

### Genus Gonatopus

Taxon classificationAnimaliaHymenopteraDryinidae

Ljungh, 1810

Gonatopus Ljungh, 1810: 161. Type species: Gonatopus formicarius Ljungh, 1810, by monotypy.

#### Diagnosis.

Female: Apterous or macropterous; palpal formula 3/2, 4/2, 4/3, 5/2, 5/3, or 6/3; pronotum crossed or not by transverse furrow; enlarged claw with distal apex pointed and with one large or small subapical tooth (occasionally subapical tooth absent, then enlarged claw with distal group of lamellae); in macropterous forms, protarsomere V with more than 20 lamellae; tibial spurs 1/0/1. Male: Fully winged; occipital carina absent or incomplete (in this last case, present behind and shortly on sides of posterior ocelli); occiput concave; temple present; palpal formula 3/2, 4/2, 4/3, 5/2, 5/3, or 6/3; tibial spurs 1/1/2.

### Gonatopus jacki sp. n.

Taxon classificationAnimaliaHymenopteraDryinidae

http://zoobank.org/58D11919-7B91-4A07-87CA-E94B23BCE001

#### Diagnosis.

**Figure 1.**
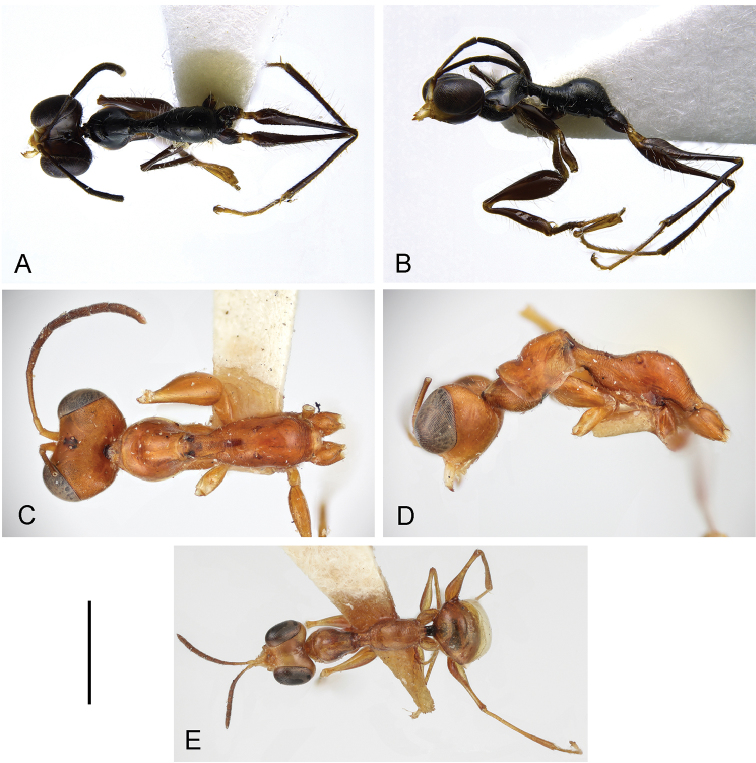
**A, B** *Gonatopus jacki* sp. n., holotype: head and mesosoma in dorsal (**A**) and lateral (**B**) view. *Gonatopus ashmeadi* holotype: head and mesosoma in dorsal (**C**) and lateral (**D**) view; *Gonatopus agropyrus* holotype in dorsal view (**E**). Scale bars: 1.38 mm (**A**);1.54 mm (**B**); 0.51 mm (**C, D**); 1.09 mm (**E**).

**Figure 2.**
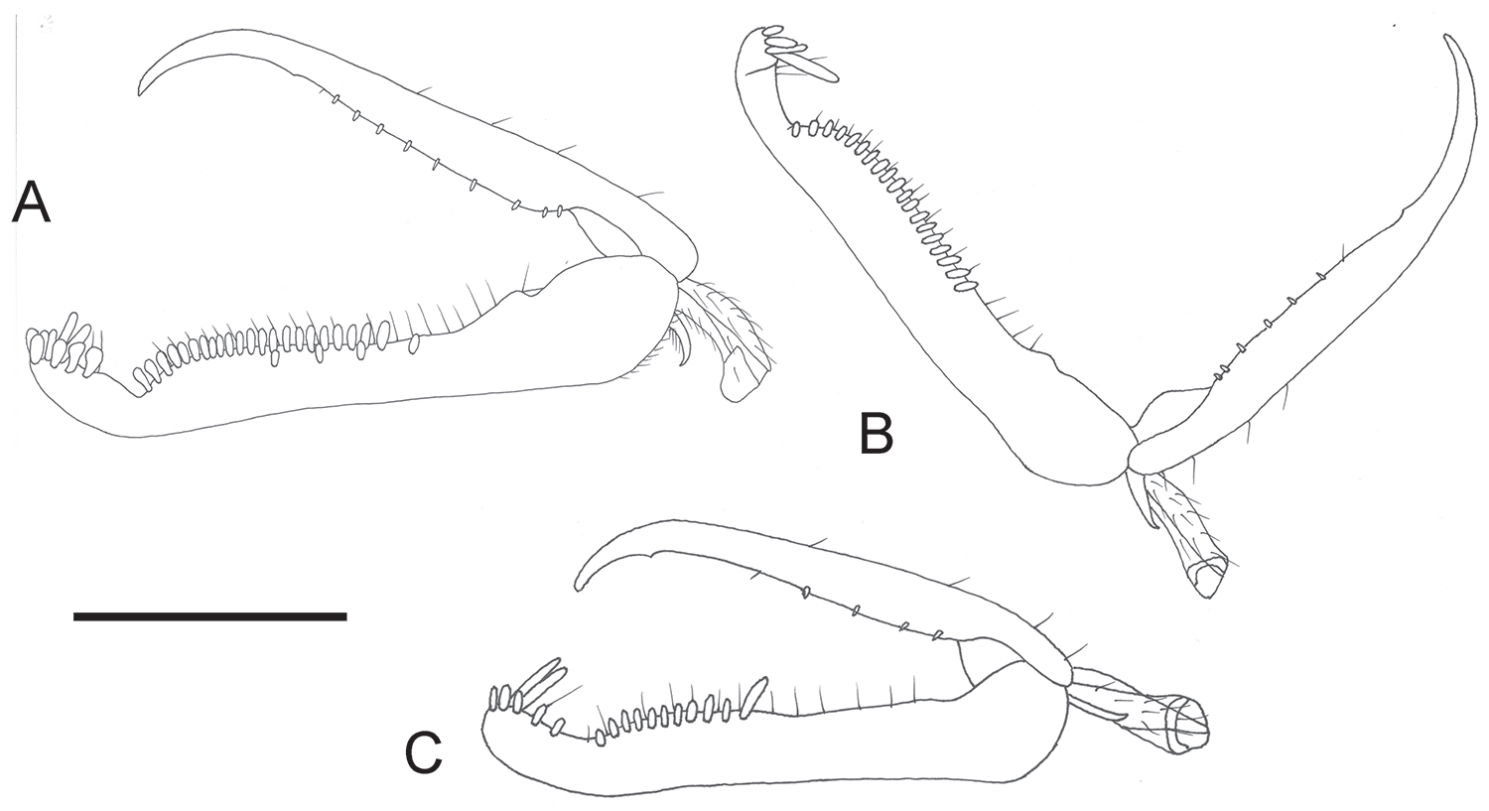
**A** *Gonatopus jacki* sp. n., chela of holotype. **B** *Gonatopus ashmeadi* Kieffer: chela of female from USA, Georgia, Spring Creek **C** *Gonatopus agropyrus* Fenton: chela of female from USA, Kentucky, Lexington. Scale bars: 0.16 mm (**A**); 0.13 mm (**B, C**).

Female apterous (Fig. 1A, B); palpal formula 5/2; pronotum crossed by deep transverse furrow (Fig. 1B); mesoscutum without lateral pointed apophyses (Fig. 1A); metanotum not hollow behind mesoscutellum (Fig. 1B); meso-metapleural suture obsolete; first abdominal tergum strongly transversely striate (Fig. 1A); enlarged claw with peg-like hairs and one small subapical tooth (Fig. 2A).

#### Description.

***Female.*** Apterous (Fig. 1A, B); length 3.4 mm. Head brown, except mandible, clypeus, region of face between antennal toruli and two short frontal stripes along orbits yellow-whitish; antenna brown, except antennomere 10 whitish; mesosoma and metasoma black; legs brown, except metatrochanter testaceous. Antenna clavate; antennomeres in following proportions: 9:6:14:10:10:9:8:8:7:10. Head excavated, shiny, not sculptured; frontal line complete; occipital carina absent; POL = 1; OL = 2; OOL = 8; greatest breadth of posterior ocellus about as long as POL. Palpal formula 5/2. Mesosoma with long sparse setae. Pronotum shiny, unsculptured, crossed by deep transverse impression. Mesoscutum slender (Fig. 1A), dull, granulated, laterally without pointed apophyses (Fig. 1A). Mesoscutellum very small, flat, not sculptured. Metanotum flat, transversely striate, not hollow behind mesoscutellum (Fig. 1B). Metapectal-propodeal complex shiny, with metapostonotum not sculptured; first abdominal tergum transversely striate. Mesopleuron and metapleuron granulated and transversely striate. Meso-metapleural suture obsolete. Protarsomeres in following proportions: 13:2:4:17:26. Protarsomere III produced into hook. Enlarged claw (Fig. 2A) with one small subapical tooth and nine peg-like hairs + one bristle. Protarsomere V (Fig. 2A) with two rows of 4 + 22 lamellae situated in distal half; distal apex with approximately eleven lamellae. Tibial spurs 1/0/1.

***Male.*** Unknown.

#### Material examined.

**Holotype**: a female from the USA, Florida, Sarasota Co., Turtle Beach, 27.217°N 82.517°W ± 2 km, 5 m, 30.xii.1989, beach margin, No 2601-S, John T. Longino leg. (MOLC).

#### Distribution.

USA.

#### Hosts.

Unknown.

#### Etymology.

The species is named after the collector, John T. (Jack) Longino.

#### Remarks.

The new species is similar to *G. ashmeadi* Kieffer in Kieffer & Marshall, 1905 and *G. agropyrus* Fenton, 1921, by having head mostly brown, labial palpus bi-segmented, mesoscum with no lateral pointed apophyses, meso-metapleural suture obsolete, first abdominal tergum transversely striate, protarsomere I shorter than IV, protarsomere V with lamellae situated in distal half. The main difference among *G. jacki* and the other two species is in the mesoscutum shape: very slender in *G. jacki* (Fig. 1A); broader in *G. ashmeadi* (Fig. 1C) and *G. agropyrus* (Fig. 1E). The comparison of the holotypes of the above three species shows also a difference regarding the colour: mostly black in *G. jacki* (Fig. 1A, B), yellow-testaceous in *G. agropyrus* (Fig. 1E) and testaceous-ferruginous in *G. ashmeadi* (Fig. 1C, D). However, these differences are not significant, because the colour can be very variable, so that mostly black specimens are known in both *G. agropyrus* and *G. ashmeadi*. Following the description of *G. jacki*, the key to the females of the Nearctic species of *Gonatopus* group 7 published by Olmi (1993b) can be modified by replacing couplet 14 as follows:

**Table:** 

14	Scutum very slender (Fig. 1A)	***G. jacki* sp. n.**
–	Scutum broader (Fig. 1C, E)	14*
14*	Protarsomere V with lamellae of approximately same length (Fig. 2B)	***G. ashmeadi* Kieffer**
–	Protarsomere V with lamellae much longer near base (Fig. 2C)	***G. agropyrus* Fenton**

## Conclusions

Olmi (1984, 1987, 1992, 1993a, 1993b, 1995, 2003) and Olmi and Guglielmino (2013) listed 51 *Gonatopus* from the Nearctic region. With the description of the above new species the number of species now known in the USA is 52. In comparison with the 135 species listed in Mexico (Moya Raygoza and Olmi 2010), the dryinid fauna of the USA is poorly known, as is that of Canada (18 listed species). A similar situation exists also regarding the hosts: they are known only in 26 species (Guglielmino et al. 2013).

## Supplementary Material

XML Treatment for Gonatopus

XML Treatment for Gonatopus jacki
